# Developmental Differentiations of Major Maize Stemborers Due to Global Warming in Temperate and Tropical Climates

**DOI:** 10.3390/insects14010051

**Published:** 2023-01-05

**Authors:** Baptiste Régnier, Judith Legrand, Paul-André Calatayud, François Rebaudo

**Affiliations:** 1 UMR Évolution, Génomes, Comportement et Écologie, IRD, CNRS, Université Paris-Saclay, 91190 Gif-sur-Yvette, France; 2 UMR Génétique Quantitative et Évolution—Le Moulon, Université Paris-Saclay, INRAE, CNRS, AgroParisTech, 91190 Gif-sur-Yvette, France

**Keywords:** development, modeling, insects, climate change, maize stemborers, pest management

## Abstract

**Simple Summary:**

Crop pest damage is expected to increase worldwide due to global warming. However, pest insect responses to global warming are complex, and a better understanding of the impacts of future temperature changes on pest insect populations is needed to ensure food security. Maize is a particularly important crop at all latitudes, making assessment of the impact of global warming on the development of maize stemborers in temperate and tropical climates critical. Stemborers are moths whose larvae feed on maize and tunnel through stems and ears, causing direct and indirect yield losses. We used mathematical models that relate the development of insects to temperature for four species found in Europe, North America, and sub-Saharan Africa. We quantified the positive and negative impacts of temperature projected under different climate change scenarios on the immature developmental stages of the four species. We found that global warming could either be beneficial or detrimental to pest development, depending on the optimal temperature for the development of the species and climate change scenarios. These results, within their limits, help to clarify stemborers’ responses to global warming across latitudes, and show that in the long term, the development of stemborers could be altered. This alteration will result either in delayed development or accelerated development, and may consequently impact stemborer dynamics.

**Abstract:**

While many insects are in decline due to global warming, the effect of rising temperatures on crop insect pests is uncertain. A capacity to understand future changes in crop pest populations remains critical to ensure food security. Using temperature-dependent mathematical models of the development of four maize stemborers in temperate and tropical regions, we evaluated the potential impacts of different climate change scenarios on development time. While recognizing the limitations of the temperature-dependent development rate approach, we found that global warming could either be beneficial or detrimental to pest development, depending on the optimal temperature for the development of the species and scenarios of climate change. Expected responses range from null development to 1.5 times faster development than expected today. These results suggest that in the medium term, the studied species could benefit from global warming with an accelerated development, while in the long term, their development could either be delayed or accelerated, which may impact their dynamics with implications on maize cultivation.

## 1. Introduction

While insect populations are declining worldwide [1,2,3], crop losses due to pest insects are expected to increase in the future [4]. Habitat modification associated with changes in agricultural practices beginning in the 1950s is a key factor in the decline of local insect populations [5], but the increasing amount of land converted to monocultures has provided favorable environments for insect pests to thrive [6]. In addition, warmer temperatures related to climate change are expected to favor the development, growth and survival of many insect species, so that global crop losses due to insect pests could increase by 10–25% per degree Celsius [4]. However, as highlighted in a meta-analysis on pest insect responses to climate change including 31 insect species [7], there are discrepancies in pest responses to global warming, depending on the observed trait. Fluctuations in insect populations are mainly explained by variability in environmental conditions across time and space [8]. Environmental factors, such as humidity, temperature, precipitation or wind speed, can directly impact insects by affecting their development, reproduction, survival and movement speed, among other traits [8,9]. Insects are ectotherms so temperature conditions are considered to be among the main determinants of their life history traits. Thus, changes in temperature conditions as a consequence of global warming may alter the spatio-temporal dynamics of insect populations [10].

In particular, shifts in phenology, i.e., the timing of life cycle events based on environmental fluctuations, are expected (e.g., [11]) or already being observed (e.g., [12]). These shifts can be explained by certain events, such as diapause, which is typically determined by environmental cues (e.g., [13]), and by the duration of development, which is notably impacted by the temperature of the environment [14]. The latter relationship is nonlinear and generally represented by a thermal performance curve (TPC) which shows the relationship between the development rate at one life stage, i.e., the inverse of the development time, and temperature [14,15]. Development rate quantifies the fraction of development time accomplished per unit of time (usually days) at a given temperature [16]. Development rate is null below a critical thermal minimum ($CT_{min}$), from which it increases almost linearly as the temperature rises, reaching a maximum ($T_{opt}$) and then dropping to a critical thermal maximum ($CT_{max}$), above which development rate is null. The relationship between development rate and temperature can be partially represented with a linear model [17], but this approach is limited to the temperature range where development rate can be considered linear and could lead to incorrect interpretations for insects exposed to temperatures outside this range [18,19]. As a result, several nonlinear models have been proposed to characterize the entire nonlinear relationship (see reviews [20,21,22,23]). From the relationship between the development rate and temperature, together with temperature time series, species development time has been traditionally predicted with applications in pest management (e.g., [24,25]), vector-borne disease management (e.g., [26,27]) or forensic science (e.g., [28]). In the context of global warming and the availability of global circulation models [29], interest has increasingly focused on the use of thermal performance curves to study global warming’s impact on the spatio-temporal dynamics of insect populations (e.g., [11,25,30]).

The common shape of a TPC suggests that development takes place within a certain temperature range ($\left|CT_{max}-CT_{min}\right|$), which varies according to species, populations, and life stages [31]. TPC shapes differentiate thermal specialist species, specialized in a narrow thermal range, from thermal generalist species, which can develop in a wider thermal range [14]. Species with narrow thermal ranges are generally considered more vulnerable to temperature changes [31,32]. Within this framework, a correlation between latitude and vulnerability has been reported [32,33], suggesting that species living in tropical habitats are more vulnerable to temperature changes, which could be explained by smaller daily and seasonal temperature variations in their habitat [34,35]. However, temperate species with short activity periods might also be specialized to narrow thermal ranges, suggesting a similar vulnerability to rising temperature [36]. In addition, it was found that habitat temperatures in ectotherms were generally lower than the optimal temperature-maximizing performance, because individuals experience a range of temperatures, and due to the asymmetry of TPCs, a temperature higher than the optimum temperature reduces performance more than a temperature that is equally lower [37]. Given temperature variability, temperatures can exceed the optimum temperature even if the mean temperature is below that value, so a mean temperature that approaches $T_{opt}$ suggests a negative impact on performance. Optimal temperature is then an important metric for assessing the impact of rising temperatures on insect development.

**Figure 1:**
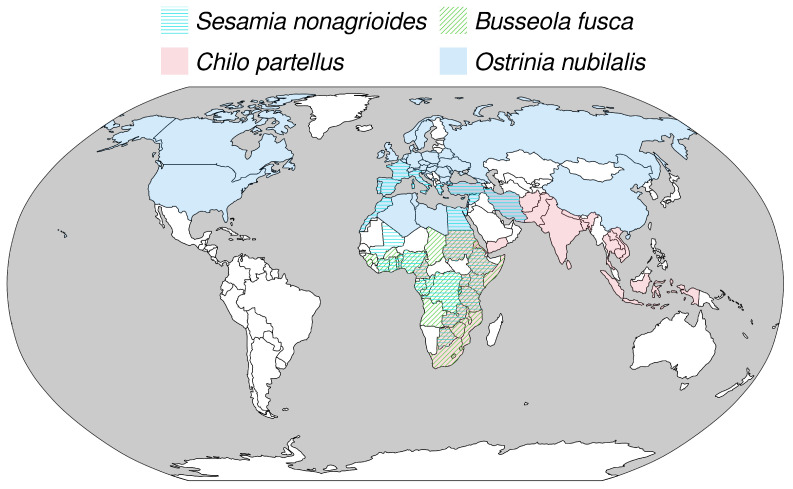
Distribution map of *Chilo partellus*, *Busseola fusca*, *Sesamia nonagrioides* and *Ostrinia nubilalis*. The map shows the countries where the presence of each species has been recorded in the literature, based on records gathered by the Centre for Agricultural Bioscience International shared under license CC BY-NC-SA 3.0 [45]. There are overlaps between the four distribution ranges, shown by the superposition of colors, particularly in Africa, where *S. nonagrioides*, *B. fusca* and *C. partellus* are found.

Maize is a cereal cultivated for human consumption and as a fodder crop, so that its production is among the largest in most regions of the world. Maize production is mainly constrained by weed competition worldwide [38], but crop losses due to animal pests, most of which are insects and mites, could reach 16% of world production in the absence of crop protection [38]. In particular, insects are important pests in areas where farmers have limited resources and small cultivated surfaces (<2 ha) bordered by natural patches with wild host plants [39]. In sub-Saharan Africa, lepidopteran stemborers are the main limitation to increasing grain production [39] and can represent losses from 5% to 73% of potential yields [39,40]. Similarly, in Europe, lepidopteran stemborers can represent losses from 5% to 30% in the absence of control measures [41]. To better understand the potential impact of climate warming on pest insects, our objective is to assess the effect of temperature increase on the development time of maize pests through a modeling study focused on four Lepidoptera in tropical and temperate climates: the European corn borer *Ostrinia nubilalis* Hübner (Lepidoptera: Crambidae), the Mediterranean corn borer *Sesamia nonagrioides* Lefebvre (Lepidoptera: Noctuidae), the spotted stem borer *Chilo partellus* Swinhoe (Lepidoptera: Crambidae), and the maize stalk borer *Busseola fusca* Fuller (Lepidoptera: Noctuidae). These species were chosen because they are major pests of maize in tropical and temperate climates [42,43,44], and because they share similar life cycles and biology, facilitating the construction of consistent developmental models, while having their own ranges across latitudes (Figure 1). For each species, we used temperature predictions for current conditions and future conditions under two greenhouse gas emission scenarios to predict the fraction of the maximal development rate of each species reached in each month of the year, and we computed the differences between future and current conditions to quantify the positive or negative impact of global warming on species development. In all geographical areas, species will be confronted with higher temperatures as a consequence of global warming, but the impact on development varies between species, scenarios, geographical regions and months due to the non-linearity of the TPCs.

## 2. Materials and Methods

### 2.1. Biological Models

The four species (*Chilo partellus*, *Busseola fusca*, *Sesamia nonagrioides*, and *Ostrinia nubilalis*) are moths and present a holometabolous development in four phases. Although the four species are important pests of maize plants [42,43,44], they can be found on other plant species in wild habitats [44,46,47,48,49]. Eggs are laid by females on maize plants, on which larvae hatch. The larvae then feed on leaves at the youngest stages, on tassels, stems or ears. Damage, including tunnels, results in direct yield losses, or indirectly by weakening the plants, increasing their susceptibility to disease and other stressors. In temperate regions, two major pest stemborers are *O. nubilalis* and *S. nonagrioides* [41], and in sub-Saharan Africa two major stemborers are *B. fusca* and *C. partellus* [44].

*O. nubilalis* is a major pest of maize in Europe and was introduced into North America at the beginning of the 20th century [42]. The larval development is composed of five stages, and individuals enter diapause during the winter at the fifth instar inside maize residues [25]. Diapause induction is mostly controlled by temperatures, photoperiod and their interaction, but also heredity and genetic factors [25]. One to four generations per year have been observed in relation to latitudinal variations in diapause induction and termination timings [50].

*S. nonagrioides* is present in sub-Saharan Africa, from Ivory Coast to Kenya, and the species’ range extends to the Mediterranean region and the Middle East, from Spain to Iran [43]. Using mitochondrial and nuclear markers, it has been demonstrated that the European and African populations belong to the same species and that the European population originated in both west and east Africa [43]. Interestingly, this species is a major pest of maize in Mediterranean Europe and in several countries in sub-Saharan west Africa but not in east Africa, where it lives on wild host plants near wet areas [51]. In temperate regions, similarly to *O. nubilalis*, individuals of *S. nonagrioides* in the last larval instar enter diapause at the end of summer inside maize debris [13]. Diapause is controlled by temperature and photoperiod and their interaction [13]. Depending on the region, there can be from two to four generations per year [13]. In tropical regions, facultative diapause occurs for populations living in sub-Saharan Africa, especially in wild habitats [51].

*C. partellus* is an invasive species in sub-Saharan Africa that was introduced from Asia during the 1930s and first reported in Malawi [52]. Since then, its presence has been reported in most of the eastern and southern African countries, and the species is essentially found in hot lowland areas at mid-altitude [53,54]. The larval development includes six larval instars, and the last instar can enter a facultative diapause during dry seasons, which ends with the return of rainy conditions [44,55].

*B. fusca* is native to sub-Saharan Africa and has a similar life cycle to *C. partellus* with six larval instars, and facultative diapause during dry seasons [44]. Unlike *C. partellus*, *B. fusca* is found in higher altitudes and in more humid and cooler areas [54]. Their last instars can also enter a facultative diapause during dry seasons and terminate with the return of rainy conditions [56].

### 2.2. Thermal Performance Curves

The relationship between developmental rate and temperature is usually characterized using experimental data. These data are obtained through experiments in which individuals or groups of individuals are reared at constant temperatures, and the time taken to reach a given life stage is measured. In this study, data on mean development times at different constant temperatures were manually extracted from published studies. Table 1 describes the references from which the data were extracted, together with the experimental designs of each study (i.e., the species considered, the region where the insects were sampled, the temperatures tested, and the life stages). Because development time for each larval instar was not described separately in all studies, we pooled the developmental times of the larval stage into one life stage to approximate the development time of the complete larval stage. We computed the inverse of mean development times to obtain the development rates.

We refer to the model fits that quantify the relationship between temperature and development rate as Thermal Performance Curves (TPC). To characterize each species’ TPC, we fitted mathematical models for the developmental rate for each life stage to the collected data. More than 30 models have been proposed in the literature to characterize the relationship between temperature and development rate [23]. We selected eleven models that allow the computation of optimal temperature for development $T_{opt}$, and critical minimum $CT_{min}$ and maximum $CT_{max}$ thresholds (Table 2). Linear models were excluded from this analysis as they do not include these threshold values and do not allow us to characterize nonlinear development rate at temperatures outside the range of those typically observed in the species habitat [17,23]. The optimal temperature for development was computed as the local maximum between $CT_{min}$ and $CT_{max}$.

For each species and each life stage, we pooled the extracted data from different studies (Table 1). For each species and each life stage, the eleven mathematical models were fitted to the pooled data using the nonlinear least squares method (NLS) and the Levenberg–Marquardt algorithm [73,74]. We then compared and selected model fits using statistical criteria and biological assumptions [75]. For statistical criteria, we used Akaike’s information criterion [76] to quantify the goodness-of-fit. Next, we categorized model fits according to the difference between each AIC value and the lowest AIC value, noted $\Delta_{AIC}$. We discarded fits with $\Delta_{AIC}\geq 10$, and we considered that fits with $\Delta_{AIC}\leq 2$ had equivalent goodness-of-fit (see [77]). For biological assumptions, we discarded fits estimating a $CT_{min}$ lower than 0 °C and/or a $CT_{max}$ higher than 50 °C, as development of the species considered in these studies are inhibited by thermal stress at these temperatures [24,42,57,62]. Lastly, we selected the fit with the lowest AIC value to select the most parsimonious fit among those filtered through the biological assumptions. As a result, we obtained one model adjustment and associated parameter estimates for each life stage of each species. We verified that the NLS assumptions of homoscedasticity and normally distributed measurement errors were validated for all model fits through graphical analysis of residuals.

We made the assumption that species’ response to temperature could be characterized by aggregating data from different experiments, while acknowledging that there may be variability in experimental designs, or between populations in different regions [78]. In addition, the NLS procedure prevents the addition of random factors, so that we could not control for a study effect in the pooled data sets. However, we checked the absence of a study effect on mean development rates in each study with ANOVAs for the four species to verify that we were able to relate development rate to temperature for each life stage of the four species, and predict individual development times at different constant temperatures.

Three development rate models were selected overall from the eleven based on AIC and biological assumptions (briere1_99, kontodimas_04, and perf2_11; Table 3). Among the eleven fits, no models were excluded based on an AIC difference higher than 10, suggesting no important differences in goodness-of-fit between model adjustments. We selected adjustments with $\Delta_{AIC}\leq 2$. The best models were then selected after discarding models with outliers in thermal thresholds. Complete descriptions of AIC and biological trait values are available on a GitHub repository (https://github.com/bapt-regnier/stemBorerCC, accessed on 2 December 2022), and estimated parameters for every selected model fit are presented in Table 3.

### 2.3. Temperature Scenarios

To study the impact of global warming on pest development, we retrieved temperature data to quantify development rates in current and future conditions, using the development models. We retrieved temperature data from the IPCC Working Group I Interactive Atlas [79,80], which provides the mean monthly temperatures predicted by multiple global circulation models (GCM) for historical climate (35 GCMs) and two scenarios for the future, SSP1-2.6 (32 GCMs) and SSP5-8.5 (34 GCMs). SSP1-2.6 is a scenario whereby global warming remains below 2 °C relatively to 1850–1900, with zero net CO_2_ emissions after 2050, and is described as a low-emission reference scenario. SSP5-8.5 is a scenario without supplementary climate policies, where CO_2_ emissions nearly double from present levels by 2050, and corresponds to a high-emission scenario. Each scenario is described in depth in Chapter 1 of the IPCC WGI report [81]. The IPCC WGI Interactive Atlas provides the monthly mean temperatures aggregated over the reference regions defined for the IPCC 6th Assessment Report [81,82] for each available GCM. We determined a region for each species based on the location in the studies from which development rate data were extracted (Table 2). Because *S. nonagrioides* individuals were collected in Greece, Spain and Morocco, we used temperature data for the Mediterranean region (MED). For *O. nubilalis*, as individuals were collected in North America, we used temperature data for the East-North America region (ENA). For *C. partellus* and *B. fusca*, as individuals were collected in Kenya and South Africa, we used data for the South-Eastern Africa region (SEAF). To obtain temperature data representative of current and future conditions, we computed the mean of each month of the year over the period 1990–2014 for current conditions, and 2081–2100 for future conditions, for each region, and each GCM projection.

To assess the impact of increasing temperatures on pest development, we used mean monthly temperatures projected by Global Circulation Models under different scenarios. The temporal scale of the temperature projections we used does not represent daily fluctuations in temperature. While there is an increase in mean temperatures, we have no information about the increase in temperature fluctuations that could also impact performance negatively [83]. When average temperatures for broader temporal scales (e.g., monthly mean temperature) approach the optimal temperature, average temperatures for narrower temporal scales (e.g., daily mean temperature) can be expected to fluctuate within a range overlapping the optimum temperature and approaching the maximum critical threshold, resulting in increased development times or null development, together with the associated stress for the species [84]. Although a higher temporal resolution would have allowed for finer predictions, monthly average temperatures allowed us to draw general conclusions about the impact of warming temperatures.

Complete temperature datasets were retrieved from the IPCC WGI Interactive Atlas GitHub repository (https://github.com/IPCC-WG1/Atlas, accessed on 11 October 2022).The entire process, from TPC fitting to development time predictions and temperature data treatment, was completed using R version 4.2.1 [85], the devRate package [86] for TPCs and individual-based models, along with the targets package [87] to ensure result reproducibility.

### 2.4. Simulations of Development Times

**Figure 2:**
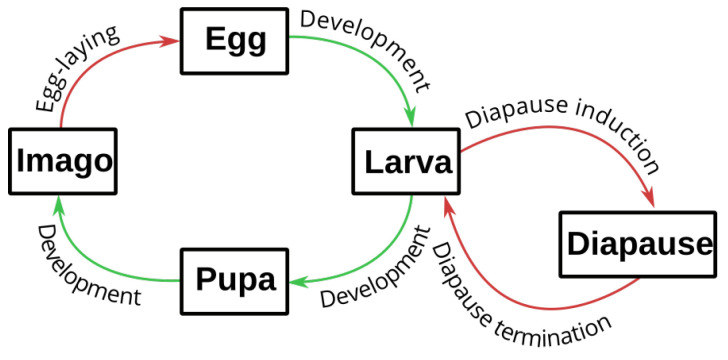
Schematic representation of the life cycle of the four maize stemborers we focused on in this study (*Chilo partellus*, *Busseola fusca*, *Sesamia nonagrioides*, *Ostrinia nubilalis*). The four species are Lepidoptera with a holometabolous development in four stages (egg, larva, pupa and imago). The number of generations per year varies between species and between populations of each species. We focused on the development of immature stages of a theoretical generation with no assumptions about its start date, corresponding to egg-laying. We made the assumption that all eggs were laid on the same date and simulated the development of individuals from egg to imago (in green) at different constant temperatures. The four species present an obligatory or facultative diapause that we did not include in the simulation study. The processes between each life stage are shown with arrows. The processes that were modeled in this work are shown in green, and those that were ignored are shown in red.

To assess the impact of temperature increase on pest development, we built an individual-based development model for each species. The model design was based on the three fitted TPCs corresponding to the egg, larval and pupal life stages of each species. The model predicts the development time of immature stages corresponding to the time required to complete the development of all stages from egg-laying to the end of the pupal stage (i.e., egg, larva and pupa) at a constant temperature *T* (Figure 2). It accounts for inter-individual variation in development rates. For each individual *i*, the development time of each life stage *j* was computed as the inverse of the individual development rate $r_{ij}\left(T\right)$, and the time from egg-laying to imago stage $d_{i}\left(T\right)$ was computed as the sum of the development time of the three life stages.

Several methods have been proposed to account for variance in development rates [88,89,90]. Here, we made the assumption that the development rate $r_{ij}\left(T\right)$ followed a normal distribution with a mean equal to the development rate $\tau_{j}\left(T\right)$ given by the TPC of each life stage *j*, and a standard deviation proportional to the mean [88], using a constant coefficient of variation cv. In the absence of data on the variance of development rates for all the species considered in this study, we assigned the value cv=0.15 based on results on *Anthonomus grandis* (Coleoptera: Curculionidae) and *Pseudatomoscelis seriatus* (Hemiptera: Miridae) [88]. A similar constant of coefficient of variation was also found for *Colaphellus bowringi* (Coleoptera: Chrysomelidae) [91]. The coefficient of variation cv was assumed constant for all three stages and all four species, so that $r_{ij}\left(T\right)∼N\left(\mu =\tau_{j}\left(T\right),\;\sigma =0.15\times \tau_{j}\left(T\right)\right)$. The values of development rate $r_{ij}\left(T\right)$ were drawn in the range $0\leq r_{ij}\left(T\right)<\infty$ to avoid negative values [88], since development rate cannot be negative.

For each species, we simulated the time required to reach the imago stage for 5000 individuals, and we checked that this number allowed the mean estimates to be reproduced. We focused on the development of immature stages of a theoretical generation and made the assumption that all eggs were laid on the same date. We computed the mean development times together with the prediction interval at 95%, at every temperature between 0 °C and 50 °C. We then quantified the temperature thresholds above or below which complete development is unattainable for half the individuals. To do so, we computed the temperatures at which 50% of the individuals could not complete their development within a time period shorter than the growing season of the host plant (estimated to be 6 months or 182 days). This duration corresponds to the time between two dry seasons in sub-Saharan Africa, and to the time from spring to autumn in higher latitudes. We refer to these two metrics as $T50_{min}$ and $T50_{max}$. We also computed the development rate for the complete immature development as the inverse of the mean development time to complete the three life stages, for all temperatures between 0 °C and 50 °C. Next, we computed the maximum development rate for complete development which we noted as $r_{max}$. It has been reported that development time can only be accurately predicted using a high temporal resolution of temperatures fluctuations [92] and by summing rates predicted at each temporal step [16]. However, we focused on the development time of a hypothetical generation at a given constant temperature, so that we could assess the impact of climate warming on pest development with monthly temperatures predicted by GCMs.

In each region and for each GCM projection, we predicted the average development rate of the species for each month, and divided this value by the maximum development rate of the species, giving a new metric noted as $r/r_{max}$, which corresponds to the fraction of maximal development reached at a given temperature. Thus, the closer the temperature is to $T_{opt}$, the closer this metric is to 1. When temperatures predicted null development rates for one of the three life stages, the metric was equal to 0. For each month and each GCM projection we computed the difference between the $r/r_{max}$ predicted with future temperatures and the same metric predicted with current temperatures. The resulting value, which we refer to as the $r/r_{max}$ difference, corresponded to a value between −1 and 1 (or −100% and +100%), and quantified the positive or negative impacts of warming temperatures on species development. For each month of the year, we computed the mean $r/r_{max}$ difference computed for all GCM projections.

## 3. Results

### 3.1. Thermal Performance Curves

**Figure 3:**
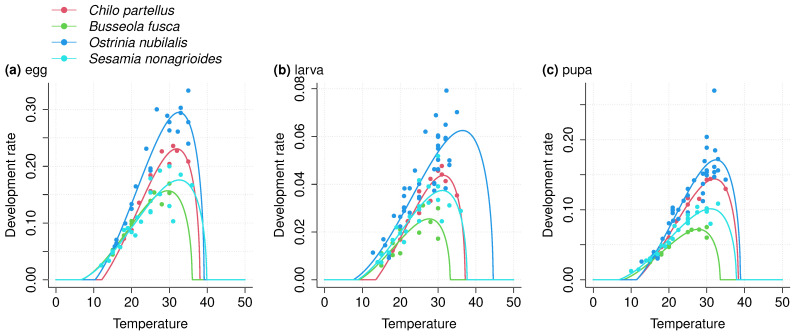
Thermal performance curves for (**a**) egg, (**b**) larva and (**c**) pupa life stages of *Chilo partellus*, *Busseola fusca*, *Ostrinia nubilalis*, and *Sesamia nonagrioides*. Development rate as a function of temperature is shown. Development rate is defined as the inverse of development time and quantifies the fraction of development time accomplished per day at a given temperature. For each species and each life stage, we extracted mean development times measured at different constant temperatures from other studies (see Table 1). We computed the inverse and pooled the extracted data from different studies for each species and each life stage. Then, eleven non-linear models were fitted to the pooled experimental data using the non-linear least squares method, and one model was selected based on statistical and biological criteria, for each species and each life stage.

**Figure 4:**
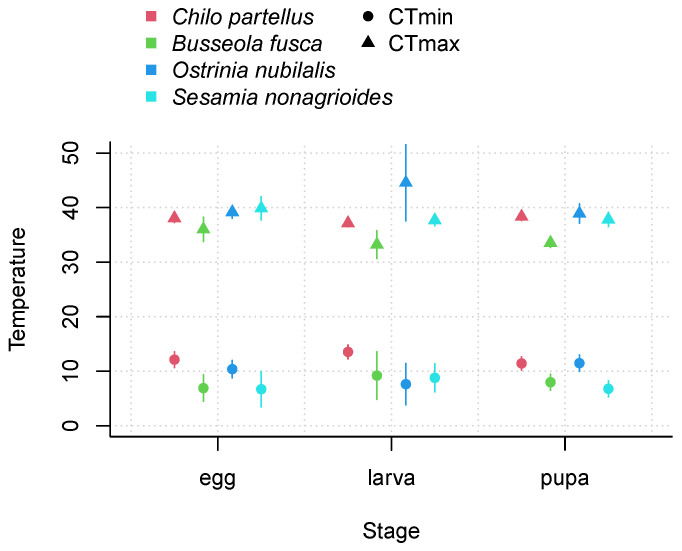
Estimated critical threshold values (±Standard Errors) for egg, larva and pupa stage of *Chilo partellus*, *Busseola fusca*, *Ostrinia nubilalis*, and *Sesamia nonagrioides*.

We observed differences in the estimated values of critical thresholds between the life stages of each species and between species, specifically in high temperatures. We observed higher values of $CT_{max}$ for the egg and larval stages of *O. nubilalis* and *S. nonagrioides* than of *C. partellus* and *B. fusca* (Figure 3 and Figure 4). However, these differences must be interpreted in light of the precision of estimates, which was generally low. Notably, standard errors of $CT_{max}$ estimates were high (Table 3; Figure 4), especially for the larval stage of *O. nubilalis* ($CT_{max}=44.2\pm 6.0$) and the egg stage of *S. nonagrioides* ($CT_{max}=46.7\pm 5.0$). For the four species and the three life stages, optimal temperature for development varied from 27.7 °C for the larval stage of *B. fusca* to 35.0 °C for the larval stage of *O. nubilalis*.

### 3.2. Impact of Temperature on Development Time

**Figure 5:**
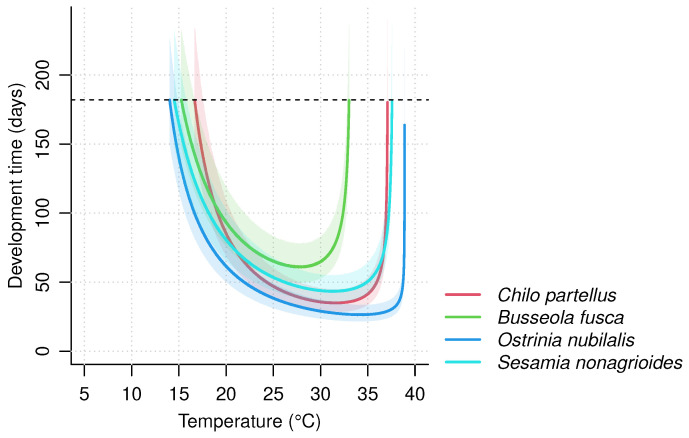
Impact of temperature on the total development time of *Chilo partellus*, *Busseola fusca*, *Ostrinia nubilalis*, and *Sesamia nonagrioides*. The development time was predicted for 5000 individuals by summing the inverse of development rates of each life stage. The development rates were drawn in a normal distribution with $\mu =\tau_{j}\left(T\right)$ and $\sigma =0.15*\tau_{j}\left(T\right)$, where $\tau_{j}\left(T\right)$ is the development rate predicted by the TPC of each life stage *j*. Mean development times as a function of temperature for the four species are represented. The 95% prediction interval is shown in transparency. The dashed line represents the 182-day limit for median development times that we fixed for characterizing the minimal and maximal temperature thresholds of each species.

Using the selected models, we predicted the complete development time of 5000 individuals for each of the four species, at all temperatures between 0 °C and 50 °C. Development time as a function of temperature varied between the four species (Figure 5). The temperature thresholds above or below which 50% of the individuals could not complete their development within 182 days ($T50_{min}$ and $T50_{max}$) and consequently the thermal range ($T50_{max}-T50_{min}$) varied between the four species. *B. fusca* had the narrowest thermal range ($T50_{min}=15.2$ °C; $T50_{max}=33.0$ °C) followed by *C. partellus* ($T50_{min}=16.6$ °C; $T50_{max}=37.1$ °C). *S. nonagrioides* and *O. nubilalis* had the two greatest thermal ranges, with, respectively, $T50_{min}$ equal to 14.4 °C and 14.0 °C, and $T50_{max}$ equal to 37.6 °C and 38.9 °C.

### 3.3. Impacts of Warming Temperatures

**Figure 6:**
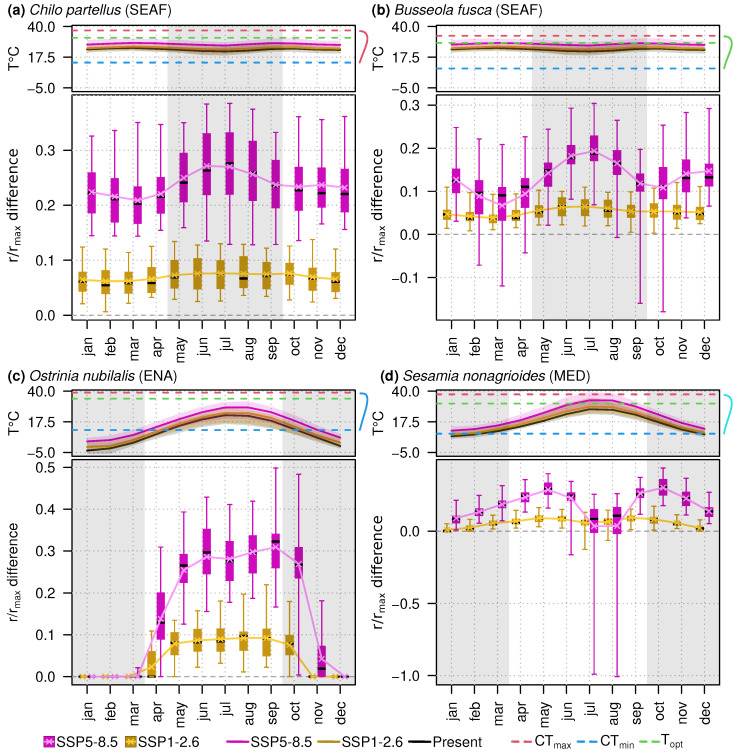
Influence of global warming on the development of (**a**) *Chilo partellus*, (**b**) *Busseola fusca*, (**c**) *Ostrinia nubilalis*, and (**d**) *Sesamia nonagrioides*. For each species, we predicted average development rates using mean monthly temperatures predicted by multiple Global Circulation Models (GCM) under current conditions (N = 35), and two future scenarios, SSP1-2.6 (N = 32) and SSP5-8.5 (N = 34), averaged over the region of reference where the species is present (*C. partellus* and *B. fusca*: South-Eastern Africa, SEAF; *O. nubilalis*: East-North America, ENA; *S. nonagrioides*: Mediterranean region, MED). For each GCM, we averaged the monthly temperatures over 1990–2014 to represent current conditions, and 2081–2100 for future conditions. Monthly temperatures for each scenario are represented in the upper part, together with the area between minimal and maximal temperatures in transparency, and the species’ total development $CT_{min}$ (blue dashed line), $T_{opt}$ (green dashed line), $CT_{max}$ (red dashed line), and the development rate as a function of temperature in the right-hand part. The predicted development rate divided by the maximal development rate of the species, noted $r/r_{max}$, was computed for all temperature predictions, and the difference between the value predicted in each future scenario and the value predicted in current conditions is represented as a function of the months of the year in the lower part. The mean differences are shown with with pink/yellow crosses and solid lines. The value where no difference is observed in $r/r_{max}$ between current temperatures and future temperatures, i.e., 0, is represented as a gray dashed line. Values above this line translate into an increase in development rate in the future in comparison with current conditions, and inversely the values below 0 represent a decrease in development rate in the future. Gray areas correspond to periods of the year when the species are not expected to develop, i.e., the dry season in tropical regions where (**a**) *C. partellus* and (**b**) *B. fusca* are found, and autumn and winter in temperate regions where (**c**) *O. nubilalis* and (**d**) *S. nonagrioides* are found. The whiskers of the boxplots represent the minimal and maximal values.

In south-east Africa, where *C. partellus* and *B. fusca* are found, the two species will be confronted with higher monthly temperatures than in current conditions, leading to both increased and decreased development rates depending on the species and temperature predictions. For *C. partellus*, the mean value of $r/r_{max}$ differences across GCMs predictions varied from 6.2% to 7.6% between months under SSP1-2.6, and from 20.8% to 27.2% under SSP5-8.5 (Figure 6a). The $r/r_{max}$ differences under SSP5-8.5 were always greater than under SSP1-2.6 for all GCMs across the year. For *B. fusca*, the mean $r/r_{max}$ difference varied between months and was not always greater under SSP5-8.5 than under SSP1-2.6. Especially in February, March, and April, during the rainy season when maize is grown and the species is expected to develop, the development rate could decrease relatively to current conditions depending on the GCM predictions (Figure 6b). Six GCMs predict under SSP5-8.5 that monthly temperatures could be higher than the optimal temperature of *B. fusca* (Figure 6b), leading to a prediction of the average development rate lower than the average development rate predicted with current temperatures.

In north-east America, *O. nubilalis* will be confronted with higher temperatures than today, leading to increases in development rate during the maize growing season, with a greater extent under SSP5-8.5 (from 13.8% in April to 30.9% in September) than under SSP1-2.6 (from 2.3% in April to 9.2% in August) (Figure 6c). All GCM projections predict increases in temperature within the linear zone of the species TPC, which lead to greater average development rates (Figure 6c). Although the species is expected to be in diapause during October and November, we observed that warming temperatures resulted in the prediction of a positive development rate, while in current conditions the predicted average development rate was null.

In the Mediterranean region where *S. nonagrioides* is found, the mean of the differences in $r/r_{max}$ under current and future conditions predicted under SSP5-8.5 is always positive over the maize growing season, and varied from 3.5% in July to 28.8% in May (Figure 6d). However, for 8 of the 32 GCM projections, July and August temperatures should be above the $T_{opt}$ of the species, leading to a decrease in the development rate compared to the current situation. Notably, the model MIROC6 [93] predicts temperatures above *S. nonagrioides*$CT_{max}$ in July and August, leading to a null average development rate (Figure 6d). Under SSP1-2.6, a similar pattern is observed between monthly predictions, with a slightly lower mean difference during July and August, respectively, 6% and 6.7%, compared to other months of the growing season with mean differences that varied from 9.1% in May to 9.4% in September, excluding April with 6.9% (Figure 6d). Under SSP1-2.6 the interquartile range in July and August did not overlap 0, as opposed to SSP5-8.5, under which the impact on development remains uncertain (Figure 6d).

## 4. Discussion

To predict the immature development time of four maize stemborers, we adjusted mathematical models to relate development rate and temperature for each life stage of each species, using nonlinear regressions on aggregated average development rate data measured at constant temperatures extracted from the literature. We estimated higher $CT_{max}$ values for the egg and larval stages of *O. nubilalis* and *S. nonagrioides*, resulting in larger thermal ranges. This is in line with previous studies that have established a relationship between latitude and thermal range, explained by the fact that species at higher latitudes are confronted with wider thermal variations [33]. In this study, the two species with greater thermal range are found at higher latitudes, i.e., *S. nonagrioides* and *O. nubilalis* [42,43], while *C. partellus* and *B. fusca* are found exclusively in the tropical regions of sub-Saharan Africa [44]. However, the standard errors of parameter estimates characterizing the TPC of each life stage showed that uncertainty remains, particularly for the larval stage of *O. nubilalis*, for which the standard error of the $CT_{max}$ estimate was the greatest, due to the lack of experimental data in higher temperatures. In addition, this parameter is likely to be overestimated, as a modeling study on the development rate of *O. nubilalis* found a lower value for $CT_{max}$ (40.9±0.2 °C, [94]) using a model from [95].

Using temperature projections from an ensemble of GCMs, we found that warming temperatures could negatively affect *S. nonagrioides* development time. Maximum daily temperature will be greater than the mean monthly temperature, so that the negative impact on the development of *S. nonagrioides*, facing temperatures above their optimal temperatures, could be greater than those predicted, especially when the frequency and intensity of heatwaves are projected to increase most [81]. Our results suggested a similar negative effect on development time for *B. fusca*, as temperature projected under SSP5-8.5 may overlap its optimal temperature for development. In contrast, a modeling study on *B. fusca* spatio-temporal dynamics under global warming predicted an increase in the number of generations [96]. However, temperature projections for the year 2055 were used [96], while we used projections for the period 2080–2100, suggesting that the species may benefit from temperature warming in the medium term but that its developmental time may be negatively affected in the long term. For the two other species, mean temperatures are always found in a range lower than the optimal temperature, where development rate increases linearly with temperature, suggesting a positive effect on development rate proportional to the increase in temperature. These results are in accordance with other findings on the future spatio-temporal dynamics of *C. partellus* and *O. nubilalis* [25,96]. However, habitat temperatures of ectotherms are generally found in suboptimal temperatures [37], suggesting that increases in mean temperatures closer to the optimal temperature could lead to daily fluctuations in ranges where development rates temporarily decrease.

Despite the expectation that tropical species would be more vulnerable to climate [32], we did not find clear evidence to support this result based on the four species. In South-East Africa, *B. fusca* could be more vulnerable than *C. partellus*, as temperatures may rise above its optimal temperature. Yet, the two species currently live at different altitudes [54], and while *B. fusca* is usually found in higher altitudes with wetter and colder conditions, *C. partellus* is found in lower altitudes with a drier and hotter environment. The spatial scale that was used for characterizing the habitat temperatures of the species could not allow us to take into account such differences in the microclimates of the species. As with *B. fusca*, *S. nonagrioides* is expected to be more vulnerable in the Mediterranean region than *O. nubilalis* in the North-East America region. However, both regions are currently characterized by different climatic conditions, as the Mediterranean region has a drier and hotter climate than the North-East America region. As a consequence, the habitat temperatures in the Mediterranean region are currently closer to *S. nonagrioides*’ optimal temperature than it is the case for *O. nubilis* in North-East America, which explains the higher vulnerability of *S. nonagrioides*. In addition, on the scale of the Mediterranean region, there are differences in climatic conditions between the north and south of the region, and changes are expected to differ along this gradient [97], which are not accounted for using temperatures averaged over the entire region. Still, the geographical range of *S. nonagrioides* could expand to northern latitudes [98], and our results on temperature-dependent development time suggest that the temperature conditions in the Mediterranean region may be disadvantageous in the future, which could provide additional evidence for the expected shift in geographical range. Over the Mediterranean region, however, it has been acknowledged that GCMs can predict more warming in summer than regional circulation models [99], so the negative impacts on *S. nonagrioides*’ development may be lower than we predicted. In addition, the four species spend most of their development time as larvae, tunneling through maize stems, where temperatures may depart from near-surface air temperature. The spatial resolution of temperature projections limits the interpretation of the impact of higher temperatures on pest development, bearing in mind that temperature conditions can differ vastly even on the scale of a plant leaf [100]. Further knowledge about temperature variations on the scale of an insect are needed to better understand the impact of global warming [101]. In the case of a maize field, temperatures could differ outside and inside the field, as maize plants provide shade [101], but also outside and inside a stem, since stems could act as an insulator. It is unclear whether temperatures experienced by stemborer larvae are lower than those measured and predicted for near-surface air, which would imply lower impact on pest development than those we predicted.

Our approach using physiological rates to assess the impact of temperature warming ignores several mechanisms that could buffer the impact of climate change. The complexity of microclimatic mosaics results in microhabitats that could either buffer or magnify the impact [101], and changes in areas of repartition could allow species to develop in favorable conditions, e.g., the shift to northern latitudes of *S. nonagrioides* [98] or the competitive displacement to higher altitudes of *B. fusca* by *C. partellus* [53]. In addition, we observed that *S. nonagrioides*’ development in July and August could be affected, while being accelerated in earlier months, suggesting that the temporal dynamics might shift compared to current observations, as reported for other Lepidoptera species (e.g., [12]). Furthermore, we ignored possible changes in agricultural practices and the potential impact of climate change on maize cultivation, which could indirectly influence pest development [102,103,104]. Finally, our modeling approach ignores several other traits, such as mortality, which is also temperature-dependent [14], as well as diapause induction and termination, which could be modified through changes in winter conditions in temperate regions, with a complex impact on the rest of the life cycle [105].

To implement inter-individual variation in the simulation study, we made the assumption that the coefficient of variation was independent from temperature. However, this assumption may have limitations when mortality at high and low temperatures reduces variability [88] or when diapause is induced by a certain temperature threshold in all individuals, leading to a reduced variance in development times (e.g., [106,107]). If our assumption that inter-individual variance is proportional to the mean development rate at a constant temperature is true, we can expect larger variances in low and high temperatures, suggesting that individuals with the lowest development times in these temperatures would have an evolutive advantage over others. It has been reported that extreme temperatures influence the evolution of insects’ thermal tolerance [108], so that evolutive adaptations could buffer the negative impact of climate change, as discussed for other ectotherms, such as amphibians and reptiles [109]. However, the general lack of knowledge about the inter-individual variability of development times in insects led us to make restrictive assumptions [88].

Despite the limits of our approach, we observed that the development time of maize pests may be altered depending on the regions considered, the species’ response to temperature, and the climatic scenario for the future. While our results may not be generalized to other pest species, the methodological approach using temperature-dependent developmental rate models could be extended to other species, given the abundance of experimental data published in the literature [23]. If overall greater damage to crops is expected with temperature increases [4], here we found by focusing on the temperature-dependent development of four major maize stem borers that two of these species could face temperatures above their optimal temperature for development, suggesting a negative impact on development time. If *O. nubilalis* and *C. partellus* will most probably benefit from higher temperatures in their respective regions, the positive impact on *S. nonagrioides* and *B. fusca* is more uncertain. Pest responses to global warming differ depending on the observed traits [7]. Here, we focused on development time and found contrasting responses between species, given the complex nature of the nonlinear development response to temperature of the species studied. In addition, we used temperature projections from an ensemble of GCMs under two greenhouse gas emission scenarios, revealing the uncertain impact on development time given the uncertainty of possible temperature changes. Still, warming temperatures will have an impact on the development time of stemborers, and consequently lead to changes in their spatio-temporal dynamics. These changes will directly impact damage to maize crops and suggest that changes may be required in farming practices for maize cultivation.

## Figures and Tables

**Table 1:** Studies from which the data were extracted to build thermal performance curves of development rate.

Species	Region	Temperatures (°C)	Life Stages ^1^	Reference
*S. nonagrioides*	Greece	14, 17, 21, 25, 31	e, l, p	[57]
*S. nonagrioides*	Greece	20, 22.5, 25, 27.5, 30	e, l, p	[58]
*S. nonagrioides*	Spain	12, 15, 18, 21.5, 25, 27.5, 30, 33, 36	e, l, p	[59]
*S. nonagrioides*	Morocco	15, 19, 25, 30	e, l, p	[60]
*O. nubilalis*	Iowa	17, 21, 25, 29, 30, 32	l, p	[42]
*O. nubilalis*	North Dakota	17, 21, 29, 30, 32	l, p	[42]
*O. nubilalis*	Delaware	17, 21, 29, 30, 32	l, p	[42]
*O. nubilalis*	Missouri	17, 21, 29, 30, 32	l, p	[42]
*O. nubilalis*	Illinois	15.6, 18.3, 21.1, 23.9, 26.7, 29.4, 32.2, 35	e, l, p	[61]
*O. nubilalis* ^2^	Quebec	16, 20, 22.5, 25, 30, 33, 35	e, l, p	[25]
*C. partellus*	Kenya	18, 20, 25, 30, 32, 35	e, l, p	[62]
*C. partellus*	Kenya	22, 25, 28, 31	e, l, p	[63]
*B. fusca*	Kenya	15, 18, 20, 25, 28, 30	e, l, p	[64]
*B. fusca*	South Africa	15, 18, 20, 26, 30	e, l, p	[24]

^1^ e: eggs; l: larvae; p: pupae; ^2^ two strains that differ in their voltinism have been studied.

**Table 2:** Nonlinear models used to relate development rate with temperature. r(T) corresponds to development rate, *T* corresponds to temperature, *a*, *b*, *c*, $k_{1}$, $k_{2}$ corresponds to constants, $T_{opt}$ corresponds to the optimal temperature for development, $CT_{min}$ and $CT_{max}$ correspond to the minimal and maximal critical thresholds for development. Models were chosen because they allow the computation of $T_{opt}$, $CT_{min}$ and $CT_{max}$.

Name	Equation	Reference
analytis_77	$r\left(T\right)=a{\left(T-CT_{min}\right)}^{b}{\left(CT_{max}-T\right)}^{c}$	[65]
ratkowsky_83	$r\left(T\right)={\left[c\left(T-CT_{min}\right)\left(1-e^{k(T-CT_{max})}\right)\right]}^{2}$	[66]
hilbertLogan_83	$r\left(T\right)=\phi \left[\frac{{\left(T-CT_{min}\right)}^{2}}{{\left(T-CT_{min}\right)}^{2}+a^{2}}-\exp\left(\frac{-(CT_{max}-\left(T-CT_{min}\right))}{\Delta_{T}}\right)\right]$	[19]
beta_95	$r\left(T\right)=e^{\mu }{\left(T-CT_{min}\right)}^{a}{\left(CT_{max}-T\right)}^{b}$	[67]
beta_16	$r\left(T\right)=r_{m}\left(\frac{CT_{max}-T}{CT_{max}-T_{opt}}\right){\left(\frac{T-CT_{min}}{T_{opt}-CT_{min}}\right)}^{\left(\frac{T_{opt}-CT_{min}}{CT_{max}-T_{opt}}\right)}$	[68]
briere1_99	$r\left(T\right)=aT\left(T-CT_{min}\right){\left(CT_{max}-T\right)}^{\left(1/2\right)}$	[69]
briere2_99	$r\left(T\right)=aT\left(T-CT_{min}\right){\left(CT_{max}-T\right)}^{\left(1/b\right)}$	[69]
kontodimas_04	$r\left(T\right)=a{\left(T-CT_{min}\right)}^{2}\left(CT_{max}-T\right)$	[70]
shi_11	$r\left(T\right)=c\left(1-e^{-k_{1}\left(T-CT_{min}\right)}\right)\left(1-e^{k_{2}\left(T-CT_{max}\right)}\right)$	[71]
perf2_11	$r\left(T\right)=c\left(T-CT_{min}\right)\left(1-e^{k(T-CT_{max})}\right)$	[71]
regniere_12	$r\left(T\right)=\phi \left[e^{b(T-CT_{min})}-\left(\frac{CT_{max}-T}{CT_{max}-CT_{min}}\right)e^{-b\left(T-CT_{min}\right)/\Delta_{b}}-\left(\frac{T-CT_{min}}{T_{m}-CT_{min}}\right)e^{b\left(CT_{max}-CT_{min}\right)-\left(CT_{max}-T\right)/\Delta_{b}}\right]$	[72]

**Table 3:** Selected model for each life stage of *Chilo partellus*, *Busseola fusca*, *Ostrinia nubilalis* and *Sesamia nonagrioides*, and their estimated parameter values with standard errors. For all models, $T_{opt}$ was estimated as the local maximum between $CT_{min}$ and $CT_{max}$ for models impeding analytical computation.

Species	Stage	Model	Parameter	Estimate	Standard Error
*Chilo partellus*	egg	kontodimas_04	*a*	$4.7\times 10^{-5}$	$1.2\times 10^{-5}$
			$CT_{min}$	10.4	1.2
			$CT_{max}$	42.3	1.5
			$T_{opt}$	31.6	
	larva	briere1_99	*a*	$3.3\times 10^{-5}$	$4.2\times 10^{-6}$
			$CT_{min}$	13.5	1.3
			$CT_{max}$	37.1	0.6
			$T_{opt}$	31.4	
	pupa	briere1_99	*a*	$8.7\times 10^{-5}$	$9.8\times 10^{-6}$
			$CT_{min}$	11.4	1.2
			$CT_{max}$	38.3	0.7
			$T_{opt}$	32.1	
*Busseola fusca*	egg	kontodimas_04	*a*	$2.2\times 10^{-5}$	$9.1\times 10^{-5}$
			$CT_{min}$	5.9	1.7
			$CT_{max}$	42.2	3.7
			$T_{opt}$	30.0	
	larva	briere1_99	*a*	$2.1\times 10^{-5}$	$1.0\times 10^{-5}$
			$CT_{min}$	9.2	4.3
			$CT_{max}$	33.2	2.6
			$T_{opt}$	27.7	
	pupa	briere1_99	*a*	$5.5\times 10^{-5}$	$8.4\times 10^{-6}$
			$CT_{min}$	8.0	1.5
			$CT_{max}$	33.5	0.8
			$T_{opt}$	27.8	
*Ostrinia nubilalis*	egg	kontodimas_04	*a*	$3.3\times 10^{-5}$	$1.2\times 10^{-5}$
			$CT_{min}$	8.0	1.7
			$CT_{max}$	46.8	3.4
			$T_{opt}$	33.8	
	larva	briere1_99	*a*	$2.1\times 10^{-5}$	$7.6\times 10^{-6}$
			$CT_{min}$	7.9	3.2
			$CT_{max}$	44.2	6.0
			$T_{opt}$	35.0	
	pupa	perf2_11	*c*	$8.8\times 10^{-3}$	$8.3\times 10^{-4}$
			*k*	0.92	1.0
			$CT_{min}$	11.8	1.1
			$CT_{max}$	36.3	1.5
			$T_{opt}$	33.0	
*Sesamia nonagrioides*	egg	kontodimas_04	*a*	$1.6\times 10^{-5}$	$8.7\times 10^{-6}$
			$CT_{min}$	5.6	2.8
			$CT_{max}$	46.7	5.0
			$T_{opt}$	32.9	
	larva	kontodimas_04	*a*	$5.6\times 10^{-6}$	$1.6\times 10^{-6}$
			$CT_{min}$	7.8	1.5
			$CT_{max}$	41.9	1.7
			$T_{opt}$	30.5	
	pupa	briere1_99	*a*	$1.2\times 10^{-5}$	$3.3\times 10^{-6}$
			$CT_{min}$	5.7	1.3
			$CT_{max}$	44.0	2.6
			$T_{opt}$	31.0	

## Data Availability

Publicly available datasets were analyzed in this study. All results of this study can be reproduced using the Github repository (https://github.com/bapt-regnier/stemBorerCC, accessed on 2 December 2022 ) under a GPL v3 licence. The scripts on this repository use R version 4.2.1 and the targets package version 0.13.5 to ensure reproducibility.
